# Bacteria Isolated From Patients With Cholelithiasis and Their Antibacterial Susceptibility Pattern

**DOI:** 10.5812/ircmj.3883

**Published:** 2013-08-05

**Authors:** Mohammad Moazeni Bistgani, Reza Imani

**Affiliations:** 1 Department of Surgery, Faculty of Medicine, Shahrekord University of Medical Sciences Shahrekord, IR Iran; 2 Department of Infectious Diseases, Faculty of Medicine, Shahrekord University of Medical Sciences, Shahrekord, IR Iran

**Keywords:** Antibacterial Susceptibility Pattern, Bacteria, Cholelithiasis

Dear Editor,

The most prevalent complication of cholelithiasis is chronic cholecystitis usually necessitating cholecystectomy (1). The biliary tract is usually sterile; however, if cholelithiasis is pigmented and cholestering, different microbes might be identified in and/or cultured from the bile or gallbladder wall (2). Microscopic examinations indicated that 20-50% of the patients with chronic cholecystitis have positive bile culture (3). Different reasons for biliary tract infection have been presented, e.g. ascending infection due to reflux of duodenal contents, blood-borne infection and infection spread through the portal-venues channels. Ascending infection from the duodenum is thought to be the primary mechanism by which bacteria enter the bile (4). Different microbes in the bile may be cause to post- cholecystectomy infections. Thus, understanding the most common organisms causing them and their antibacterial susceptibility pattern would be useful in prevention of these infections. The present study was carried out to achieve this aim.

In this cross sectional study, 132 patients with cholelithiasis and chronic cholecystitis without risk factors for postoperative sepsis were subjected to elective laparoscopic cholecystectomy from September 2009 to September 2010. We ordered antibiotic prophylaxy routinely with 1gr keflin one house before operation. Using a questionnaire, patients' age, sex, clinical features of the patients, the isolated bacteria, and antibiogeram were recorded. During surgery 5-10 ml of the bile samples were aspirated with sterile syringe from gallbladder immediately after cholecystectomy; 3-5 ml was placed in a sterile container; another 3-5 ml was inoculated directly into an aerobic and anaerobic BacT/Alert blood culture bottle. The Samples were sent immediately to the Laboratory and incubated at 37°C for 24h. After the bacteria were isolated, we performed antibiotic sensitivity tests by the isolates carried out using Kirby-Bauer method with a colony in Mueller-Hinton agar medium (pH 7.2-7.4). The results were reported as susceptible, resistant and intermediate based on the diameter of the clear zone around disks with reference to the antibiotic standard table. The Data were analyzed by Chi-Square test and P<0.05 was considered significant.

From the total 132 bile samples, bacteria were isolated in 50 samples (37.87%). Some other studies have shown that 20-50% of the patients with chronic cholecystitis have positive bile culture (3). Mean age of patients with positive and negative cultures was 56.9 ± 14 and 52.7 ± 14.7 years respectively, having significant difference based on t student test (P < 0.05). A study by Al Harbi et al. has shown being older than 50 years was the only factor significant in view of preoperative positive bile culture (5), which is in agreement with our study. Anaerobic bacteria were detected in 8 (16%), Monomicrobial infection in 47 (94%), and Polymicrobial infection in 3 (6%) patients. In a study by Ballal et al. in India, bile cultures for aerobic and anaerobic bacteria were carried out on 125 samples from patients with chronic cholecystitis with cholelithiasis; 71 (56.8%) aerobic and 17 (13.6%) anaerobic bacteria were detected. Among the mixed flora, 2 had only aerobes and the remaining 5 had both aerobes and anaerobes (6). Al Harbi et al.'s study also showed Polymicrobial infection in 4 (3.57%) and anaerobic bacteria in none of the cases (5). Anaerobic bacteria may grow when the bile duct is seriously infected by anaerobic and aerobic bacteria and hence body's immunity becomes low. Lou et al. suggested the paucity of anaerobes in the human biliary system (7). The effect of anaerobic bacteria on bile pigment stone has been reported widely (8), according to which the anaerobes such as *B. fragilis* and *B. fusiformis* may produce an *E. coli* substance called β-Lactamase which resolves bilirubin. The bilirubin when integrated with calcium ion forms calcium bilirubinate.

The difference in anaerobic and Polymicrobial positive culture between our study and others' could be attributed to the method of antibiotic therapy; here, we ordered antibiotic prophylaxy routinely while in Ballal et al.'s study it was selectively and in Al Harbi et al.'s study we had not known.

In our study *E. coli* was the most common isolate (13; 26%), as previously reported (9). Enterobacter was the second one (9; 18%) followed by Salmonella Typhi (7; 14%), Coagolase-negative *staphylococcus* (6; 12%), *Klebsiella pneumoniae* (2; 4%) and *Proteus* (2; 4%). The significance of *E. coli* dominance is also supported by Maki's studies (1966) indicating a potential role for *E. coli's* Glucosonidase enzymatic activity in formation of calcium bilirubinate gall stone (10). In our study, Salmonella Typhi grew in 18% cases, sensitive to clindamicin and novobiocine in 88.8%. The prevalence of Salmonella Typhi in bile of cholelithiasis patients varied widely from 1% to 34% (11), perhaps due to typhoid fever which is, similar to some parts of our country, endemic in some regions. Sexwise analysis of our patients showed that Salmonella Typhi, more common in females compared to males, was isolated only from samples of female patients, reported elsewhere. Its high incidence in females has been attributed to hormonal effects related to menstrual cycle and pregnancy (6). Eslami et al.'s work conducted in Iran indicated *E. coli* (25%) as the most prevalent isolated bacterium, and the prevalence of *Klebsiella*, *Aerobacter*, *Pseudomonas*, *Enterococci*, and *Proteus* as 12%, 10%, 9%, 8%, and 3% respectively (12). In Ballal et al.'s study E.coli and Klebsiella were predominant among the aerobes with 45.07% and 25.35% prevalence respectively and *B. fragilis* (58.82%) predominant among the anaerobes (6). In the study of Al Harbi et al. the most common organisms isolated were *E. coli* (26.1%), *Enterococcus facials* (15.6%), and *Pseudomonas aeroginosa* (9.6%) (5). we don’t know why our findings were not completely similar to others'. However, we support taking cultures of the bile at cholecystectomy because appropriate antibiotics can be administered in case of cultures being positive, hence avoiding serious complications, e.g. gram negative septicemia. In our study *E. coli* was 92.3% susceptible to Amikacin and Enterococci 69.23% susceptible to Cephtriaxone. The most susceptible antibiotic for microorganisms on the whole was Amikacin. Ballal et al. have shown anaerobes were sensitive to Cefotaxime, Metronidazole, Chloramphenicol, Cefazolin, and tetracycline, and aerobes isolated to Ampicillin, Chloramphenicol, streptomycin, tetracycline, Gentamicin, and second generation Fluoroquinolones such as ciprofloxacin and Norfloxacin (6).

In spite of the fact that we ordered antibiotic prophylaxy for all of the patients, bacterial isolates and infectious complications showed no significant difference. Thus, we recommend starting antibiotics selectively, if supported by the clinical conditions and/or culture reports, in case of cholecystitis and cholelithiasis. However, routine culture of all bile samples is strongly advised.

Given our and others' studies anaerobes are rare in the human biliary system; therefore, if antibiotic therapy is considered, aerobic coverage should be satisfactory. In addition because the most susceptible antibiotic for microorganisms on the whole was Amikacin, this antibiotic can be started selectively.
